# Prediction model for cardiovascular events or all-cause mortality in incident dialysis patients

**DOI:** 10.1371/journal.pone.0221352

**Published:** 2019-08-22

**Authors:** Daijo Inaguma, Daichi Morii, Daijiro Kabata, Hiroyuki Yoshida, Akihito Tanaka, Eri Koshi-Ito, Kazuo Takahashi, Hiroki Hayashi, Shigehisa Koide, Naotake Tsuboi, Midori Hasegawa, Ayumi Shintani, Yukio Yuzawa

**Affiliations:** 1 Department of Nephrology, Fujita Health University School of Medicine, Toyoake, Japan; 2 The Aichi Cohort Study of Prognosis in Patients Newly Initiated into Dialysis (AICOPP) Group, Aichi, Japan; 3 Department of Medical Statistics, Osaka City University Graduate School of Medicine, Osaka, Japan; 4 Department of Nephrology, Nagoya University School of Medicine, Nagoya, Japan; International University of Health and Welfare, School of Medicine, JAPAN

## Abstract

Some variables including age, comorbidity of diabetes, and so on at dialysis initiation are associated with patient prognosis. Cardiovascular (CV) events are a major cause of death, and adequate models that predict prognosis in dialysis patients are warranted. Therefore, we created models using some variables at dialysis initiation. We used a database of 1,520 consecutive dialysis patients (median age, 70 years; 492 women [32.4%]) from a multicenter prospective cohort study. We established the primary endpoint as a composite of the incidence of first CV events or all-cause death. A multivariable Cox proportional hazard regression model was used to construct a model. We considered a complex and a simple model. We used area under the receiver operating characteristic curve (AUROC) to assess and compare the predictive performances of the prediction models and evaluated the improvement in discrimination using the complex model versus the simple model using net reclassification improvement (NRI). We then assessed integrated discrimination improvement (IDI) to evaluate improvements in average sensitivity and specificity. Of 392 deaths, 152 were CV-related. Totally, 506 CV events occurred during the follow-up period (median 1,285 days). Finally, 692 patients reached the primary endpoint. Baseline data were set at dialysis initiation. AUROC for the primary endpoint was 0.737 (95% confidence interval [CI], 0.712–0.761) in the simple model and 0.765 (95% CI, 0.741–0.788) in the complex model. There were significant intergroup differences in NRI (0.44; 95% CI, 0.34–0.53; p < 0.001) and IDI (0.02; 95% CI, 0.02–0.03; p < 0.001). We prepared a Shiny R application for each model to automatically calculate the predicted occurrence probability (https://statacademy.shinyapps.io/App_inaguma_20190717/). The complex model made more accurate predictions than the simple model. However, the intergroup difference was not significant. Hence, the simple model was more useful than the complex model. The tool was useful in a real-world clinical setting because it required only routinely available variables. Moreover, we emphasized that the tool could predict the incidence of CV events or all-cause mortality for individual patients. In the future, we must confirm its external validity in other prospective cohorts.

## Introduction

The number of patients with end-stage kidney disease (ESKD), which causes social, medical, and socioeconomic issues, is increasing worldwide. As of December 31, 2016, there were 726,331 (2,128 per million population) cases of ESKD in the U.S. and 329,609 (2596.7 per million population) cases in Japan. Of them, 124,675 cases in the U.S. and 39,344 cases in Japan were newly reported [1, 2]. Moreover, the number of elderly patients or those with comorbid cardiovascular (CV) diseases or low cognitive function is expected to increase annually. CV disease accounted for 48% of deaths among dialysis patients in the U.S. [1]. Dialysis patients at high risk of CV disease and all-cause death require more intensive management including blood pressure, anemia, and mineral metabolism management. Moreover, although renal replacement therapy (RRT) is required when kidney function declines due to ESKD, dialysis initiation or kidney transplantation does not always lead to improved quality of life in all ESKD patients [3–5]. In other words, we must decide to start RRT from various viewpoints. Hence, it is helpful to be able to estimate survival rates or the incidence of CV events of each individual after the start of RRT.

Accordingly, shared decision-making (SDM) regarding the start of RRT is conducted in some countries. In Japan, Watanabe et al proposed an SDM process regarding the initiation and continuation of maintenance hemodialysis [6]. SDM for dialysis initiation requires evidence that allows patients, nephrologists, and medical staff derive appropriate information about prognosis including CV events. Some previous reports showed that variable clinical factors were related to mortality or CV events [7–10]. In addition, prognostic prediction score models were invented and examined. Thamer et al created a scoring system and showed in a retrospective cohort study that variables including age, serum albumin, and activities of daily living at dialysis initiation were associated with mortality within 3 months after dialysis initiation [11]. Wick et al also demonstrated that similar variables were associated with mortality within 6 months after dialysis initiation [12].

We conducted a multicenter prospective cohort study in incident dialysis patients from October 2011 to September 2016 [13] that aimed to clarify whether the management of chronic kidney disease (CKD), including blood pressure, anemia, and mineral management, during the pre-dialysis period influenced prognosis after dialysis initiation. We showed that some variables including serum calcium level, serum phosphate level, and use of vitamin D receptor activators at dialysis initiation were associated with all-cause mortality or incidence of CV events in dialysis patients [14–18]. Therefore, based on these results, we created models that predicted the incidence of CV events or all-cause mortality using some variables at dialysis initiation. This study aimed to examine the accuracy and usefulness of these models.

## Materials and methods

### Subjects

We used a database from the multicenter prospective Aichi Cohort Study of Prognosis in Patients Newly Initiated into Dialysis (AICOPP), a multicenter prospective cohort analysis of 1,520 consecutive patients aged 20 years or older who started dialysis at one of 17 AICOPP group centers in Aichi Prefecture, Japan, between October 2011 and September 2013 [13]. At the 17 centers, 1,889 patients started dialysis for ESKD or acute kidney injury during the study period. We did not include 369 patients who died around the time of dialysis initiation or withdrew from maintenance dialysis. We also excluded patients who withdrew from dialysis during hospitalization or declined to provide consent.

### Demographic and clinical characteristics of study cohort

We used demographic and clinical characteristics of the study cohort to create a prediction model tool. The clinical characteristics was based on baseline AICOPP data at the time of dialysis initiation. Body mass index (BMI), blood pressure, heart rate, and other parameters were measured just before the first dialysis session. A diagnosis of coronary artery disease (CAD) was based on information taken from the medical records. A history of CAD was defined as a history of percutaneous coronary artery intervention or coronary artery bypass graft, ischemic change seen on electrocardiogram with symptoms including chest pain on exertion, positive findings on stress myocardial scintigraphy or non-ST elevation myocardial infarction. A history of ischemic or hemorrhagic type was defined as hospitalization for stroke treatment or obvious positive findings on diagnostic imaging, including computed tomography and magnetic resonance imaging. According to American Diabetes Association criteria [19], we defined diabetes as the following: a fasting blood glucose level ≥ 126 mg/dL, random blood glucose level ≥ 200 mg/dL, hemoglobin A1c (National Glycohemoglobin Standardization Program) level ≥ 6.5%, use of insulin, or use of oral hypoglycemic agents. Before the first dialysis session, blood samples were taken for laboratory testing and a chest X-ray was performed to evaluate cardiothoracic ratio and aortic calcification. The following formulas were used to calculate estimated glomerular filtration rate (eGFR) by sex: for males, eGFR (mL/min/1.73 m^2^) = 194 × [age]^-0.287^ × [serum creatinine (mg/dL)]^-1.094^; and for females, eGFR (mL/min/1.73 m^2^) = 194 × [age]^-0.287^ × [serum creatinine (mg/dL)]^-1.094^ × 0.739 [20]. Information about medication was also obtained from the medical records. Medication use referred to drugs the patients were taking before dialysis and at the time of dialysis initiation.

### Endpoint and definition of CV events

We established primary endpoint as composite of incidence of first CV events or all-cause death. CV events were defined as heart failure requiring hospitalization, acute coronary syndrome, stroke, or peripheral artery disease requiring hospitalization. Heart failure was defined as hypoxemia and pulmonary congestion, pulmonary edema, or pleural effusion on chest radiography. Acute coronary syndrome was defined as stenosis or occlusion on coronary angiography or by percutaneous coronary intervention, coronary artery bypass surgery, electrocardiogram findings consistent with acute coronary syndrome, or non-ST elevation myocardial infarction. Stroke was defined as the presence of neurological symptoms plus brain computed tomography or magnetic resonance imaging findings indicative of hemorrhage or infarction. Survival prognosis as of September 30, 2016 was determined from medical records. For patients who were transferred to other institutions, information was obtained using a mailed survey.

### Statistical analysis

All data indicating patients’ baseline demographic and clinical characteristics are expressed as mean and standard deviation or median and interquartile range for continuous variables and counts and percentages for categorical variables.

A multivariable Cox proportional hazard regression model was used to construct a model that predicts an occurrence probability of CV events or all-cause death at 1 year since dialysis was initiated for each patient. Here we considered a couple of prediction models (complex and simple). The complex model included the following 25 predictors: sex, age at dialysis initiation, presence or absence of diabetes mellitus, aortic calcification and use of renin angiotensin system (RAS) inhibitor, use of beta-blocker, phosphate binder and vitamin D receptor activator (VDRA), history of CAD, stroke and malignancy, Barthel Index, hemoglobin, albumin, uric acid, urea nitrogen, creatinine, eGFR, potassium, adjusted calcium, phosphorus, C-reactive protein (CRP), BMI, alkaline phosphatase, and intact parathyroid hormone. In the complex model, to improve prediction accuracy, the triple- and double-product terms between age, eGFR and urea nitrogen, and aortic calcification, and corrected calcium and phosphorus were considered. And the non-linear effects on the outcome were considered for continuous variables using restricted-cubic-spline, excluding age, BUN, calcium, and phosphorus. Furthermore, for clinically practical use, the simple model was also constructed with the following 12 predictors: sex, age, presence or absence of diabetes mellitus, use of RAS inhibitor, use of beta-blocker, history of CAD and stroke, hemoglobin, albumin, urea nitrogen, eGFR, and CRP, all of which are commonly collected in clinical situations. The triple- and double-product terms between age, eGFR and CRP, and non-linearities for all continuous variables were considered similarly to the complex model.

To assess and compare the predictive performances of the prediction models, we used area under the receiver operating characteristic curve (AUROC). We calculated the bootstrapped mean values and 95% confidence intervals of AUC with 200 iterations to validate the predictive performances. We also evaluated the improvement in discrimination using the complex and simple model by net reclassification improvement (NRI). Integrated discrimination improvement (IDI) was assessed to evaluate improvements in average sensitivity and specificity using the complex and simple model.

Moreover, we prepared a Shiny R application for each model to enable automatic calculations of the predicted occurrence probability of CV-related events or all-cause death at 1 year since dialysis initiation (https://statacademy.shinyapps.io/App_inaguma_20190717/).

All statistical inferences were made with a two-sided significance level of 5% using R software version 3.5.1 (https://www.r-project.org/foundation/) with the “rms” package.

### Ethics

This study followed the ethical guidelines for clinical research by the Japanese Ministry of Health, Labour and Welfare (created July 30, 2003; full revision, December 28, 2004; full revision, July 31, 2008) and the Helsinki Declaration (revised 2013) and was approved by the clinical research ethics committees at each AICOPP group facility (Clinical Research Ethics Committee in Center for Research Promotion and Support, Fujita Health University; approval number: 20110823–3). The subjects received oral and written explanations of the study purpose and provided written consent. The trial registration number is UMIN 7096 (registered January 18, 2012).

## Results

### Demographic and clinical characteristics of study cohort

**Fig 1:**
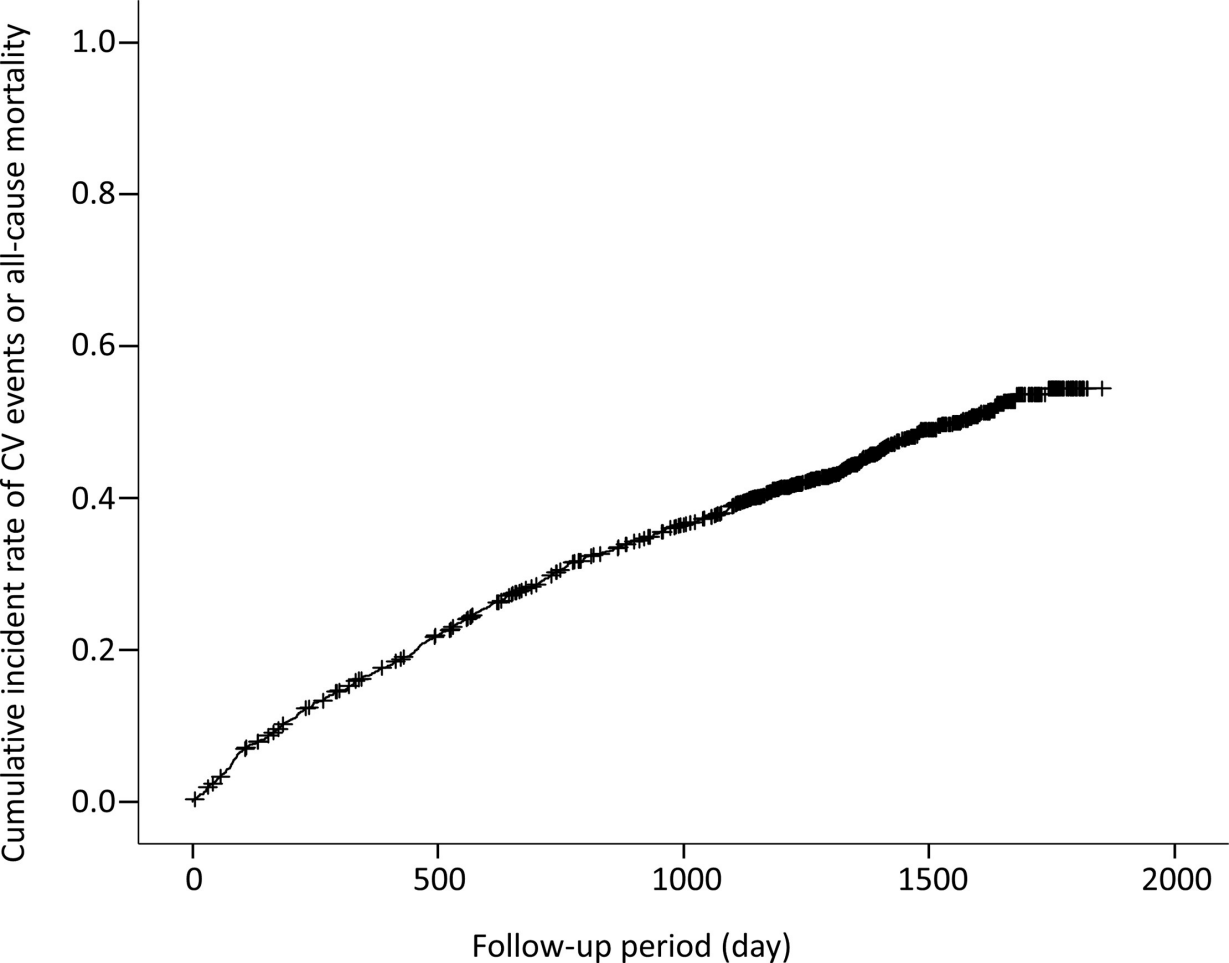
Cumulative incidence of cardiovascular events or all-cause mortality during follow-up period.

There were 392 deaths (152 CV-related) and 506 CV events during follow-up period (median 1,285 days). Finally, 692 patients reached the primary endpoints. Fig 1 shows the cumulative incident rate for primary endpoints. Table 1 shows the demographic and clinical characteristics of the study cohort by primary endpoint. Data were obtained at baseline, which was set as dialysis initiation. There were significant differences between patients who survived without CV events and patients with CV events or who died. In particular, there were more patients with diabetes, a history of CAD or stroke, and malignancy among patients with CV events or who died. Baseline kidney function was more preserved among patients with CV events or who died. Medications including RAS inhibitors, VDRA, and phosphate binders were less often used among patients with CV events or who died. The baseline raw data are shown in a supporting file (S1 Dataset).

**Table 1:** Comparison of patient characteristics and laboratory data at dialysis initiation.

Variables	Overall (n = 1,520)	Survival without CV events (n = 828)	CV events or all-cause death (n = 692)	P-value	Missing (%)
Age* (years old)	70 (60, 77)	65.5 (55, 74)	74 (65, 80)	< 0.001	0.0
Female sex (%)	492 (32.4)	306 (37.0)	186 (26.9)	< 0.001	0.0
Diabetes mellitus (%)	812 (53.4)	404 (48.8)	408 (59.0)	< 0.001	0.0
History of CAD (%)	255 (16.8)	89 (10.8)	166 (24.0)	< 0.001	0.2
History of stroke (%)	243 (16.0)	97 (11.7)	146 (21.1)	< 0.001	0.0
History of malignancy (%)	162 (10.7)	65 (7.9)	97 (14.1)	< 0.001	0.0
BMI (kg/m^2^)	23.5 (4.4)	24.0 (4.6)	22.9 (4.0)	0.008	0.0
SBP (mmHg)	151 (26)	153 (25)	149 (27)	0.047	0.9
DBP (mmHg)	77 (15)	79 (15)	74 (15)	0.130	0.9
Barthel Index*	100 (90,100)	100 (100, 100)	100 (65, 100)	< 0.001	1.7
Aortic calcification (%)	590 (38.8)	239 (29.1)	351 (50.9)	< 0.001	0.7
**Laboratory data**					
Hemoglobin (g/dL)	9.4 (1.5)	9.4 (1.6)	9.3 (1.5)	0.377	0.0
Albumin (g/dL)	3.20 (0.60)	3.26 (0.61)	3.13 (0.57)	0.110	0.9
Uric Acid (mg/dL)	8.8 (2.4)	8.7 (2.3)	8.9 (2.6)	0.044	2.2
BUN (mg/dL)	91.8 (30.5)	91.4 (30.2)	92.2 (30.8)	0.235	0.0
Creatinine (mg/dL)	8.97 (3.21)	9.51 (3.42)	8.31 (2.79)	< 0.001	0.0
eGFR (ml/min/1.73m^2^)	5.4 (2.2)	5.1 (2.1)	5.9 (2.3)	0.023	0.0
Potassium (mEq/L)	4.6 (0.8)	4.6 (0.8)	4.5 (0.9)	0.169	0.0
Adjusted calcium (mg/dL)	8.6 (1.1)	8.5 (1.1)	8.7 (1.0)	0.022	0.3
Phosphate (mg/dL)	6.4 (1.9)	6.6 (2.0)	6.2 (1.7)	0.096	1.8
Intact PTH* (pg/mL)	291 (185, 432)	308 (202, 455)	265 (164, 403)	0.014	12.5
CRP* (mg/dL)	0.30 (0.10, 1.35)	0.20 (0.08, 0.89)	0.46 (0.14, 2.07)	0.004	6.7
**Medication**					
ACEIs / ARBs (%)	917 (60.4)	529 (64.0)	388 (56.1)	0.002	0.1
beta blockers (%)	528 (34.7)	258 (31.2)	270 (39.0)	0.001	0.0
VDRA (%)	412 (27.1)	247 (29.8)	165 (23.8)	0.009	0.0
Phosphate binders (%)	532 (35.0)	344 (41.5)	188 (27.2)	< 0.001	0.0

Data are shown as mean (standard deviation), value (%), or *median (1^st^ quartile, 3^rd^ quartile) as appropriate. CV, cardiovascular; CAD, coronary artery disease; BMI, body mass index; SBP, systolic blood pressure; DBP, diastolic blood pressure; BUN, blood urea nitrogen; eGFR, estimated glomerular filtration rate; PTH, parathyroid hormone; CRP, C-reactive protein; ACEI, angiotensin-converting enzyme inhibitor; ARB, angiotensin receptor blocker; VDRA, vitamin D receptor activator

### Automatic calculation sheets

**Fig 2:**
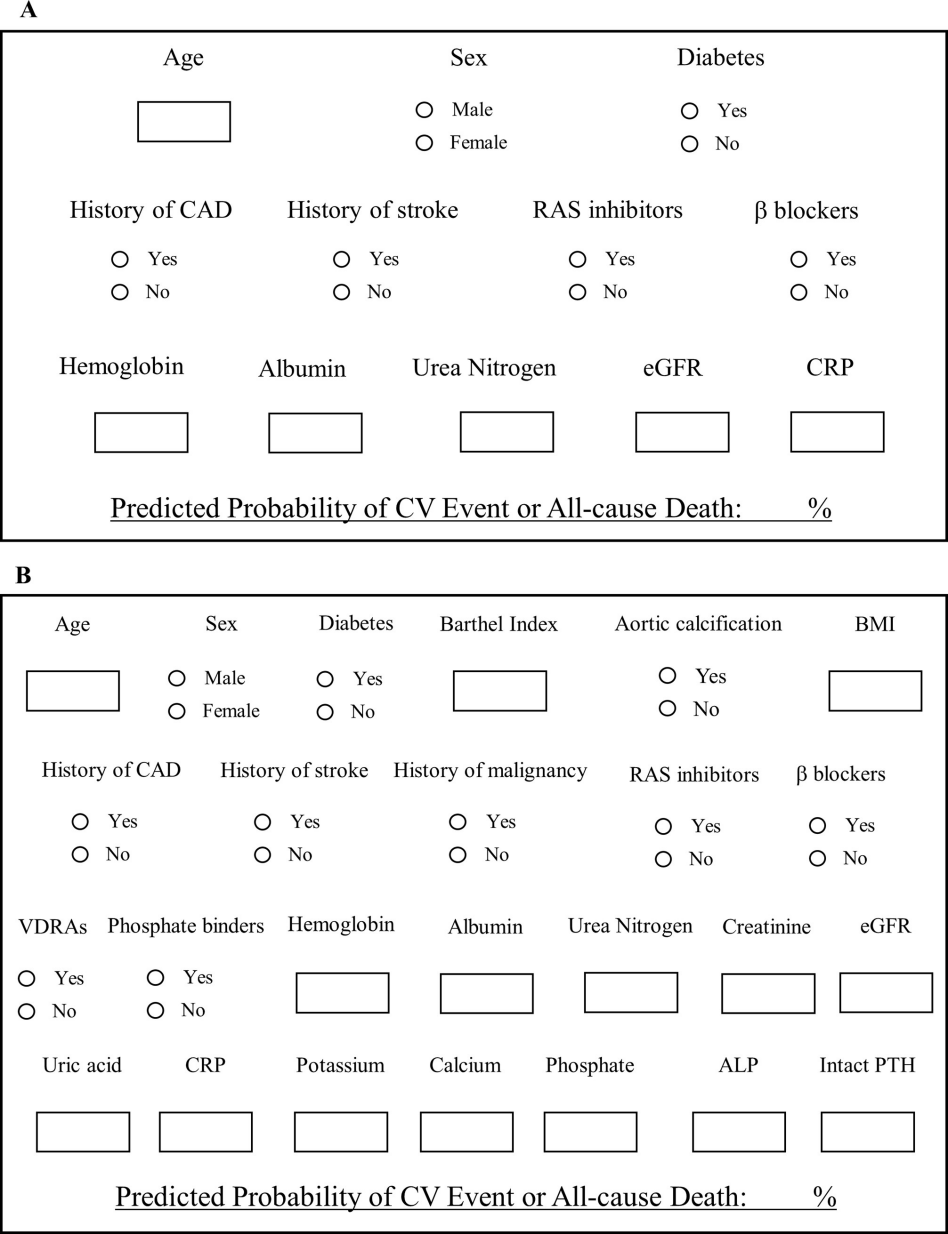
Automatic calculation sheets of predicted incidence of cardiovascular events or all-cause mortality within 1 year after dialysis initiation. **A:** simple model **B:** complex model.

Fig 2 shows the automatic calculation sheets of the predicted incidence of CV events or all-cause mortality within 1 year after dialysis initiation. Some blanks are filled with values or choice such as yes/no; thereafter, the predictive probability is automatically shown below.

### ROC curves of simple and complex models for composite endpoint

**Fig 3:**
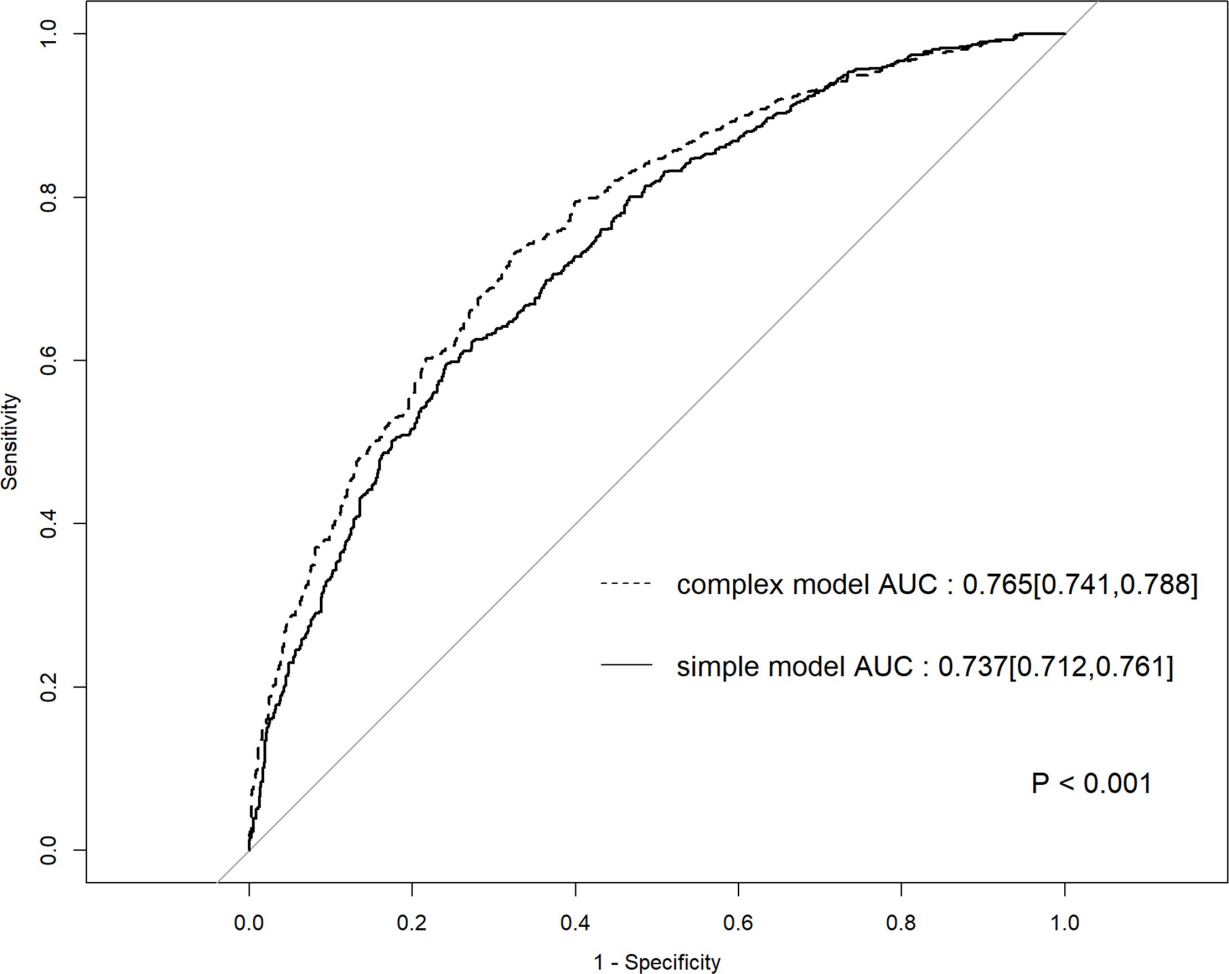
ROC curve of the simple and complex models for the composite endpoint. ROC, receiver operating characteristic; AUC, area under the curve.

**Fig 4:**
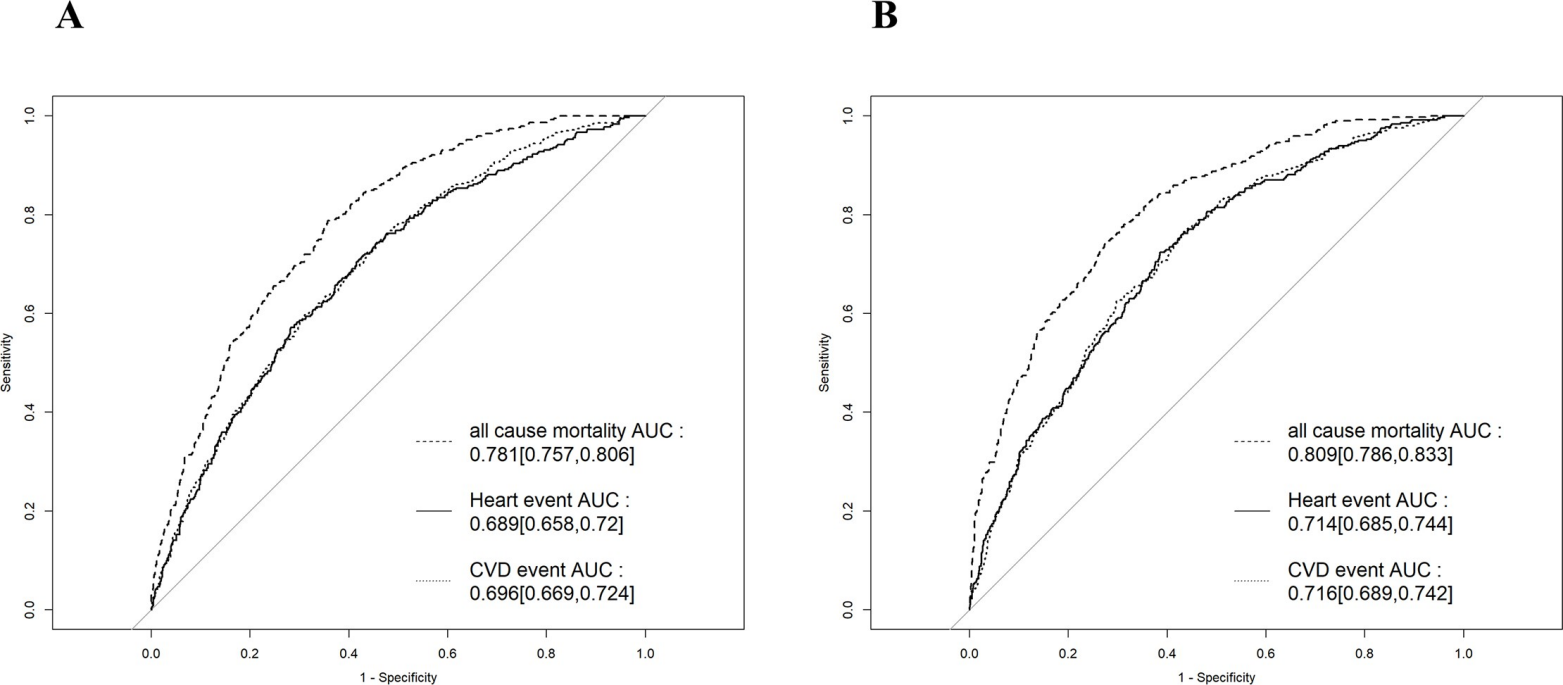
ROC curve of the simple and complex models for all-cause mortality, incidence of cardiovascular-related events, and incidence of heart disease. **A:** simple model **B:** complex model ROC, receiver operating characteristic; AUC, area under the curve.

Fig 3 shows the ROC curves of the simple and complex models for the composite endpoint. AUROC for the primary endpoint was 0.737 (95% confidence interval [CI], 0.712–0.761) in the simple model and 0.765 (95% CI, 0.741–0.788) in the complex model. There were significant intergroup differences (p < 0.001). Fig 4 shows the ROC curves of the simple and complex models for all-cause mortality, incidence of CV-related events, and incidence of CV disease separately. AUROC for the three endpoints in the simple model was 0.781 (95% CI, 0.757–0.806), 0.689 (95% CI, 0.658–0.720), and 0.696 (95% CI, 0.669–0.724), respectively. AUROC for the three endpoints in the complex model was 0.809 (95% CI, 0.786–0.833), 0.714 (95% CI, 0.685–0.744), and 0.716 (95% CI, 0.689–0.742), respectively.

### Net reclassification improvement and integrated discrimination improvement

**Fig 5:**
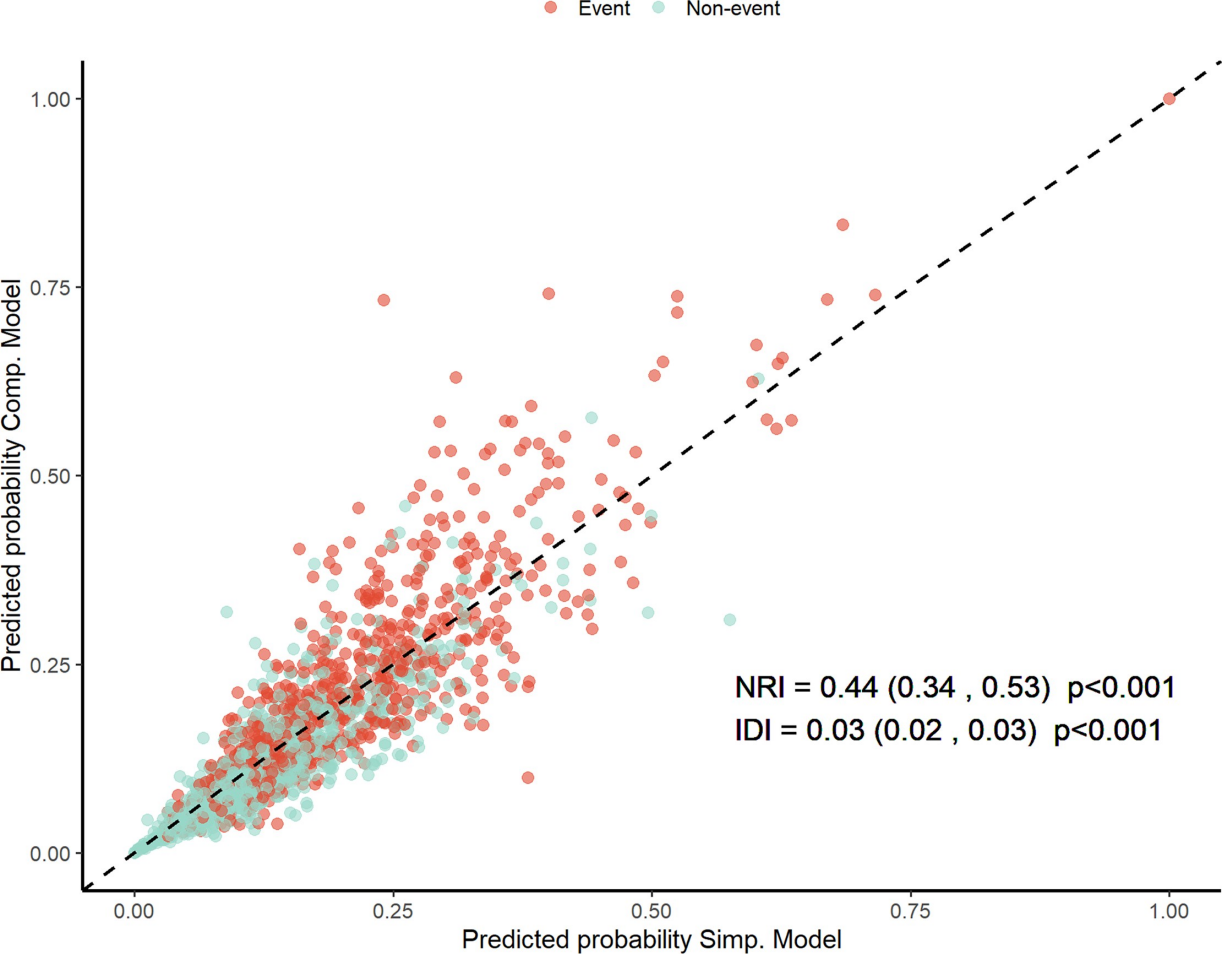
Predictive probabilities of the simple and complex models. NRI, net reclassification improvement; IDI, integrated discrimination improvement.

Fig 5 demonstrates the predictive probability of the simple and complex models. There were significant inter-model differences in NRI (0.42; 95% CI, 0.32–0.52; p < 0.001) and IDI (0.02; 95% CI, 0.02–0.03; p < 0.001).

## Discussion

Here we showed that our scoring models predicting the first incidence of CV events and all-cause mortality were worth using in clinical practice due to their accuracy and usefulness. Our models could calculate the probability of the risk for each case using more concrete numbers. It was generally recognized that mortality during early period after dialysis initiation. Therefore, we suspected that conditions present at RRT initiation would influence the incidence of CV events and mortality for at least 3 years after RRT start. It is important to predict prognosis in incident patients at the time of RRT initiation. Therefore, patients with a higher probable risk could be treated more intensively. In addition, the risk prediction tool might give information that guides the chosen timing of the start or withholding of RRT. For the above reasons, we were sure that the prediction model could be a powerful and useful tool.

Some reports described prediction models that could predict patient prognosis and mortality [11, 12, 21–23] (Table 2). Matsubara et al reported a new risk model for predicting CV events in hemodialysis patients from the database of the Japan Dialysis Outcome and Practice Patterns Study [23]. They used multivariable logistic regression with backward stepwise selection to develop a new prediction model, which showed significantly better discrimination than the Framingham risk score. Wick et al created prediction tools using logistic regression analysis and predicted early mortality among older adults after dialysis initiation [12]. Meanwhile, in our model tools, the incidence of CV events and mortality was shown as continuous values because some variables were inserted into the models as concrete figures. In other words, we can indicate risk of CV events and mortality for each patient.

**Table 2:** Previous reports of prediction models for dialysis patients.

Authors	Subjects	Number of patients	Factors used for model	outcomes	Results
Couchoud C, et al [21]	incident dialysis patients (age > 75 years)	2,500	BMI, diabetes, CHF (stages III to IV), PV disease (stages III to IV), dysrhythmia, active malignancy, severe behavioral disorder, total dependency for transfers, initial context	overall 6-month mortality	Mortality rates ranged from 8% in the lowest risk group (0 point) to 70% in the highest risk group (≥9 points) and 17% in the median group (2 points).
Thamer M, et al [11]	patients with ESRD with a previous 2-year history who initiated dialysis therapy (age > 75 years)	69,441	age, serum albumin, assistance of daily living, nursing home residence, cancer, heart failure, hospitalization	All-cause mortality in the first 3 and 6 months	the median score of 3 indicating 12% risk in 3 months and 20% in 6 months, and the highest scores ($8) indicating 39% risk in 3 months and 55% in 6 months.
Wick JP, et al [12]	incident dialysis patients (age > 65 years)	2,199	age, eGFR, atrial fibrillation, lymphoma, congestive heart failure, hospitalization in the prior 6 months, metastatic cancer	6-month mortality	a score, 5 equated to, 25% of individuals dying in 6 months, whereas a score. 12 predicted that more than half the individuals would die in the first 6 months.
Anker SD, et al [22]	maintenance hemodialysis patients	4,831	age, CV disease history, primary diabetic nephropathy, blood pressure, inflammation	2-year CV mortality	The CV mortality score was more predictive in AROii
Matsubara Y, et al [23]	Japanese maintenance dialysis patients	3,601	age, diabetes status, history of CV events, dialysis time per session, serum phosphorus, serum albumin	incidence of composite CV events and all-cause mortality	The new model showed significantly better discrimination than the FRS, in both men (c-statistics: 0.76 for new model, 0.64 for FRS) and women (c-statistics: 0.77 for new model, 0.60 for FRS)

BMI; body mass index, CHF; congestive heart failure, PV; peripheral vascular, eGFR; estimated glomerular filtration rate; CV, cardiovascular; AROii, The Analysing Data, Recognising Excellence and Optimising Outcomes Cohort; FRS, Framingham Heart Study

One feature of the current model was that the variables needed to use the tool are limited at dialysis initiation. Therefore, it is simple to decide which variables or laboratory data to use. Previous reports showed that some factors associated with prognosis including all-cause mortality in CKD patients before and at initiation of dialysis [3, 8, 22, 24–26]. In those study, baseline periods were widely ranged from CKD stage 3a to stage 5 without RRT or from incident RRT to long RRT duration. Kidney function in CKD patients usually declines and sometimes progresses to ESKD. After that, ESKD patients must continue RRT. Among those stages, RRT duration, especially for dialysis, is relatively short; therefore CKD status could be evaluated in almost the same way. Hence, we decided to choose dialysis initiation as timing of using the tool.

We would like to emphasize that all variables needed can be measured in the real-world clinical setting. In addition, only a handful of variables are needed. Hence, we consider the tool useful for general physicians as well as nephrologists. We created the model tool for predicting events after dialysis initiation. In terms of variables used for the tool, we selected demographic and those proven to be related to mortality in previous studies. We created the model tool by selecting 24 variables for the complex model because there were many variables in the database. In particular, we chose variables related to mineral and bone disorders (MBD) such as serum phosphate and parathyroid hormone level. The serum marker levels were closely associated with mortality in dialysis patients [27–30] because we were interested in the relationship between MBD parameters and mortality. We also created a simple model using 11 variables. The complex model made more accurate predictions than the simple model. However, the difference between the two versions was small. Moreover, the variables required by the simple tool are obtainable in a routine exam. Hence, we suspected that the simple model would be more useful than the complex model.

The present study had the following limitations. First, the variables used to create the prediction models were collected only at the time of dialysis initiation. In other words, it was possible that management after dialysis initiation might reflect the onset of CV events or all-cause death. Second, we did not use uniform criteria to initiate dialysis; rather, this was left to the discretion of the attending physician. Third, we did not add primary kidney disease as a predictor. The patients with chronic glomerulonephritis including immunoglobulin A nephropathy had a better prognosis because they were younger than the overall patients. We considered that predictability might be lower in the younger patients despite some variables including age being adjusted for since the number of biopsy-proved diagnoses was low.

## Conclusion

We created a new prediction model tool for the incidence of CV events or all-cause mortality after dialysis initiation. The tool will be useful in the real-world clinical setting because only routinely obtained variables were needed. Moreover, we emphasize that the tool can predict the incidence of CV events and mortality for individual patients. In the future, we must confirm the external validity in other prospective cohorts.

## Supporting information

S1 Dataset Raw data. (XLSX)
